# Etiological Spectrum and Anatomical Locations of Hydronephrosis in Western Region of Yemen

**DOI:** 10.12669/pjms.41.11.12768

**Published:** 2025-11

**Authors:** Sultan Abdulwadoud Alshoabi, Abdullatif O. Magram, Amirah F. Alsaedi, Halah Fuad Muslem, Fahad H. Alhazmi, Abdullgabbar M. Hamid

**Affiliations:** 1 Sultan Abdulwadoud Alshoabi Department of Diagnostic Radiology, College of Applied Medical sciences, Taibah University, Al-Madinah Al-Munawara, Kingdom of Saudi Arabia; 2 Abdullatif O. Magram Advanced AlRazi Diagnostic Center, Al-Hodeidah, Republic of Yemen; 3 Amirah F. Alsaedi Department of Diagnostic Radiology, College of Applied Medical sciences, Taibah University, Al-Madinah Al-Munawara, Kingdom of Saudi Arabia; 4 Halah Fuad Muslem Department of Internal Medicine, Dr. Suliman Al Habib Hospital Altakhasosi, Riyadh, Kingdom of Saudi Arabia; 5 Fahad H. Alhazmi Department of Diagnostic Radiology, College of Applied Medical sciences, Taibah University, Al-Madinah Al-Munawara, Kingdom of Saudi Arabia; 6 Abdullgabbar M. Hamid Department of Radiology, Rush University Medical Group, Chicago, IL 60612, USA

**Keywords:** Hydronephrosis, Nephrolithiasis, Vesicoureteral reflux, Pregnancy-induced hydronephrosis, Underactive bladder

## Abstract

**Background & Objective:**

Hydronephrosis is the dilatation of renal pelvis and calices with urine as due to obstruction of urine flow along the urinary tract. This study aimed to report the causes of hydronephrosis in the western region of Yemen.

**Methodology:**

This retrospective study reviewed the electronic medical records of 418 patients diagnosed with hydronephrosis between June 2024 to December 2024 at Advanced AlRazi Diagnostic Center, Al-Hodeidah, Republic of Yemen.

**Results:**

A total of 418 patients with 476 kidneys affected by hydronephrosis were included in this study. There were 265 (63.4%) male and 153 (36.6%) females, aged 1 to 80 years (mean age: 31.11±11.14). Hydronephrosis was most commonly among young adults (n=316, 66.4%). It was predominantly unilateral 361 (86.4), with right side more frequently affected than the left. The most common grade observed was Grade-II (69.7%), followed by Grade-III (17.9%). The leading cause of hydronephrosis was stones (74.4%), followed by reflux (9.5%) and pregnancy (8.8%). The stones causing hydronephrosis were most often located in the distal ureter (DU) (43.7%), followed by the middle ureter (MU) (18.5%), upper ureter (UU) (17%), vesicoureteral junction (10.5%), renal pelvis (2.3%) and urethra (0.8%). The non-parametric Kruskal-Wallis test revealed that the mean stone size increased with the grade of hydronephrosis from Grade-I to Grade-IV (p <0.001).

**Conclusion:**

This study concluded that ureteric or renal stones (nephrolithiasis) were the predominant cause of hydronephrosis, followed by reflux, pregnancy and underactive bladder. Ureteric strictures and infections were rare causes. Nephrolithiasis were commonly found in the DU, MU and UU. Grade-II hydronephrosis was the most frequently reported, with strong correlation between stone size and hydronephrosis grade, as the mean stone size increased from Grade-I to Grade-IV.

## INTRODUCTION

Hydronephrosis is a dilatation of renal pelvis and calices with urine as caused by obstruction of urine flow along the urinary tract.^1^ The Society of Fetal Urology is grading the hydronephrosis into: Grade 0: No dilatation; Grade-I: dilation of the renal pelvis; Grade-II: mild dilatation of the calices; Grade-III: severe dilatation of the calices; Grade-IV: parenchymal atrophy.^2^,^3^ The causes of hydronephrosis can be intrinsic (within the urinary tract), or extrinsic (outside the urinary tract). Both intrinsic and extrinsic causes can be congenital or acquired. Obstruction can occur at any level of the urinary tract, from the renal pelvis to the urethral orifice. Acquired intrinsic causes include stones, tumors, ureteral stricture, inflammation, infection, contracted vesical neck, urethral stricture, cyst and hypertrophy of the verumontanum.

On the other hand, congenital intrinsic causes include polycystic kidney, tumor, stone, kink, ureteral fold, ureteral valve, ureteral stricture, ureterocele, contracted vesical neck, urethral valve and urethral stricture. Extrinsic causes have a wide range from congenital anomalies such as fused, horseshoe, or cake kidney, abnormal rotation of kidney, double pelvis kidney, aneurysm of renal artery, abnormal insertion of ureter, double ureter and tumor compressing urinary tract. Acquired extrinsic causes include infection, adhesions, fibrous bands, dystrophic kidney, tumor compressing the urinary tract, ptosis, scoliosis, trauma and prostatic hypertrophy.^4^,^5^

The swift advancement and widespread use of medical imaging modalities have improved the accuracy of diagnosing hydronephrosis and identifying its underlying causes. No-contrast computed tomography (CT) is considered the gold standard imaging modality, particularly for detecting urinary tract stones.^6^ However, in most cases Ultrasonography is sufficient as the initial imaging modality due to its availability and lack of radiation exposure.^5^ When performed by an experienced operator, ultrasonography has good sensitivity and serves effectively as the first-line imaging technique in patients with renal colic. Furthermore, the presence of hydronephrosis itself enhance the sensitivity of ultrasonography in detecting ureteric stones and other obstructive causes.^7^

Despite its clinical significance, there is a lack of studies focusing on the causes of hydronephrosis, especially in Yemen. This study was conducted to explore the common causes of hydronephrosis in the Western region of Yemen. The primary aim of the study was to establish the first local reference on this topic and to compare the findings with data from different regional and international studies. The results of this study are expected to be valuable for physicians and medical students who are interested in understanding the etiologies of hydronephrosis.

## METHODOLOGY

This retrospective study was conducted using the electronic medical records of patients diagnosed with hydronephrosis between June 2024 to December 2024 at Advanced AlRazi Diagnostic Center, Al-Hodeidah, Republic of Yemen.

### Ethical approval:

This study was approved by the Institutional Review Board at Advanced AlRazi Diagnostic Center, Al-Hodeidah, Republic of Yemen (AADC 30-6-25). Due to the retrospective nature of this study, the informed consent of the participants was waived. Patients, confidentiality was strictly maintained throughout the research process.

### Study population:

Patient data were collected using a structured data collection sheet which included demographic information: age, gender and the confirmed cause of hydronephrosis. The inclusion criteria were patients diagnosed with hydronephrosis who had an available ultrasonography report determined the cause and confirmed the cause of the condition. Patients with hydronephrosis who had ultrasonography report that did not determine or confirm the cause were excluded from the study.

### Sample size:

Patients were consecutively selected during the six months period until the determined sample (n = 418) was achieved.

### Procedure:

**Fig.1:**
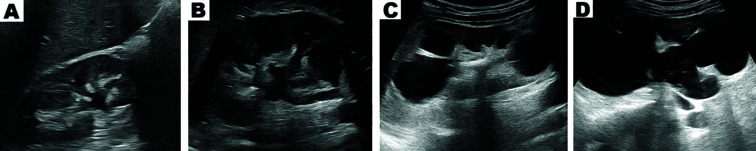
Selected ultrasonography images of grades of hydronephrosis showing: A) Grade I: dilated renal pelvis, B) Grade II: dilated renal pelvis with mild dilated some calices, C) Grade III: severe dilated calices and D) Grade IV: severe dilated calices and parenchymal atrophy.

Each case of hydronephrosis was graded (Fig.1) and the underlying cause was diagnosed. In cases where renal stones were the cause, their size was measured using ultrasonography. For patients in whom the cause of hydronephrosis could not be clearly identified with ultrasonography, additional imaging modalities such as CT or micturating cystourethrography (MCUG) were employed.

All ultrasonographic examinations were performed by a radiologist with three-years postdoctoral experience in abdominal ultrasonography, using a 3.5 MHz deep curved probe of the (Samsung Madison, Gangwon-do, Korea) ultrasound machine. All procedures were conducted in accordance with the Declaration of Helsinki, 2013 revision and followed applicable ethical standards and regulations.

### Statistical analysis:

Data analysis was carried out using SPSS version 26 (IBM, Armonk, NY, USA). Quantitative variables were presented as mean ± standard deviation, while qualitative variables were expressed as frequency and percentage. Shapiro-Wilk and Kolmogorov-Smirnov tests were performed and revealed non-normality distribution of the measurements (p < 0.001). The non-parametric Kruskal-Wallis test was used to compare the distribution of hydronephrosis by site and grades. For post hoc pairwise comparisons between hydronephrosis grades, the Dunn-Bonferroni was applied. All statistical tests were two-tailed and a p-value of less than 0.05 was considered statistically significant.

## RESULTS

A total of 418 patients with 476 instances of hydronephrosis were included and analyzed in this study. Among them, 265 (63.4%) were male and 153 (36.6%) were female, with age ranged from 1 to 80 years (mean age: 31.11±11.14 years). Hydronephrosis was most common among young adults (n=316, 66.4%), followed by children (n=49, 10.3%) and middle-aged adults (n=45, 9.5%). The majority of cases were unilateral, accounting for 361 (86.4%) of the total. Right sided hydronephrosis was more frequently observed than left sided involvement (Table-I).

**Table-I:** Sociodemographic characters of the involved patients.

Variable	Categories	Number (Percent)	P-value
Sex	Male	265 (63.4)	*P<0.001*
	Female	153 (36.6)	
	Total	418 (100%)	
Age groups	0-20	49 (10.3)
	21-40	316 (66.4)
	41-60	45 (9.5)
	>60	8 (1.7)
	Total	418 (100%)
Hydronephrosis	Unilateral	361 (86.4)	*P<0.001*
	Bilateral	57 (13.6)	
	Total	418 (100%)	
Side	Right	268 (56.3)	*P<0.001*
	Left	208 (43.7)	
	Total	476 (100%)	

The most frequently observed grade of hydronephrosis among the patients was Grade-II, found in 69.7% of cases and followed by Grade-III, which was seen in 17.9% of the cases (Table-II). The leading cause of hydronephrosis was urinary tract stones identified in 74.4% of cases. Other notable causes included vesicoureteral reflux (9.5%) and pregnancy-related hydronephrosis (8.8%) (Table-III). In term of anatomical location, the most common site of obstruction was distal ureter (DU) in 43.7%, followed by the middle ureter (MU) in 18.5%, upper ureter (UU) in 17%, vesicoureteral junction (VUJ) in 10.5%, urinary bladder (UB) in 6.1%, renal pelvis in 2.3% and urethra in 0.8% (Table-IV).

**Table-II:** Grades of hydronephrosis.

Variable	Categories	Number (Percent)	p-value
Grades of hydronephrosis	Grade-I	27 (5.7)	*P<0.001*
	Grade-II	332 (69.7)	
	Grade-III	85 (17.9)	
	Grade-IV	32 (6.7)	
	Total	476 (100%)	

**Table-III:** Causes of hydronephrosis.

Variable	Categories	Number (Percent)	p-value
Causes of hydronephrosis	Stones	354 (74.4)	*P<0.001*
	Reflux	45 (9.5)	
	Pregnancy	42 (8.8)	
	Underactive Bladder	29 (6.1)	
	Stricture	4 (0.8)	
	Infection	2 (0.4)	
	Total	476 (100%)	

**Table-IV:** Sites of causes of hydronephrosis.

Variable	Categories	Number (Percent)	P-value
Causes of hydronephrosis	Renal pelvis	11 (2.3)	*P<0.001*
	Pelviureteric junction (PUJ)	5 (1.1)	
	Upper ureter (UU)	82 (17)	
	Middle ureter (MU)	88 (18.5)	
	Distal ureter (DU)	208 (43.7)	
	Vesicoureteral junction (VUJ)	50 (10.5)	
	Urinary bladder (UB)	29 (6.1)	
	Urethra	4 (0.8)	
	Total	476 (100%)	

**Fig.2:**
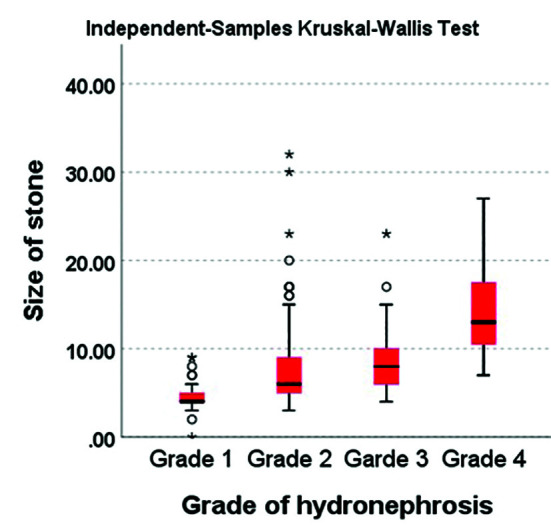
Stone size and grade of hydronephrosis.

Comparing the stone size (n=353) and the grade of hydronephrosis, using the non-parametric Kruskal-Wallis one-way ANOVA, revealed that the mean size of the stone increase as the grade of hydronephrosis increased from Grade-I to Grade-IV (Kruskal-Wallis H = 68.49, p <0.001) (Table-V, Fig.2). Pos-hoc analysis using Dunn-Bonferroni showed that all pairwise comparisons between the hydronephrosis grades, Grade-I vs. Grade-II, Grade-I vs. Grade-III, Grade-I vs. Grade-IV, Grade-II vs. Grade-III, Grade-II vs. Grade-IV and Grade-III vs. Grade-IV, were significantly different in pairs (p <0.001) (Table-VI).

**Table-V:** Correlation between size of stone and grades of hydronephrosis.

Variable	Categories	Number	Mean ± SD	Mean Rank	Chi-Square (X^2^)	p-value
Grades of hydronephrosis	Grade-I	25	4.80±2.94	81.56	43.32	P<0.001
	Grade-II	252	7.39±0.93	166.95		
	Grade-III	57	8.65±1.94	215.74		
	Grade-IV	20	14.35±3.28	312.00		
	Total	354				

**Table-VI:** Pairwise Comparisons of stone size and grade of hydronephrosis.

Sample 1-Sample 2	Test Statistic	Std. Error	Std. Test Statistic	p-value	Adjusted p-value
Grade-I-Grade-II	-85.392-	21.249	-4.019-	<0.001	<0.001
Grade-I-Garde 3	-134.177-	24.304	-5.521-	<0.001	<0.001
Grade-I-Grade-IV	-230.440-	30.395	-7.581-	<0.001	<0.001
Grade-II-Garde 3	-48.785-	14.866	-3.282-	<0.001	<0.001
Grade-II-Grade-IV	-145.048-	23.541	-6.162-	<0.001	<0.001
Garde 3-Grade-IV	-96.263-	26.332	-3.656-	<0.001	0.002

Each row tests the null hypothesis that the Sample 1 and Sample 2 distributions are the same. Asymptotic significances (2-sided tests) are displayed. The significance level is .05.

## DISCUSSION

Diagnosing of the underlying cause of hydronephrosis is essential for determining the appropriate treatment option or intervention. Early diagnosis plays critical role in effective treatment to prevent potential complications of obstructive uropathy, such as irreversible kidney damage. In the current study, we explored the spectrum of hydronephrosis causes in the western region of Yemen. We found that nephrolithiasis (kidney stones) was the leading cause of hydronephrosis, accounting for 74.4% of cases. This finding is consistent with a previous study by Thotakura and Anjum, who reported that nephrolithiasis was the most common cause of hydronephrosis in young adults.^5^ Similarly, our previous work found nephrolithiasis to be the most common obstructive cause of hydronephrosis even in children.^8^

The second most common cause identified in the current study was vesicoureteral reflux (VUR), accounting for 9.5%. VUR is primarily caused by failure of the anti-reflux mechanism in the VUJ., often due to structural and physiological anomalies in children.^9^ Updated and Summaries from the European Association of Urology and the European Society of Pediatric Urology have emphasized the importance of accurate diagnosis and classification of VUR in pediatric patients.^10^

Pregnancy was identified as the third most frequent cause in the present study, seen in 8.8% of cases. Gestational hydronephrosis is a physiological condition caused by hormonal effect and mechanical compression of the ureters by the enlarging uterus, which is commonly observed during pregnancy.^11^ It occurs in more than 80% of pregnant women and is most pronounced in primigravida. Typically, it develops during the second trimester, is more prominent in the right side and is limited to the area above the Linea terminalis (pelvic brim), usually resolving within a few weeks after delivery. The mechanical compression is caused by enlarging uterus pressing on the ureters at the point where they cross the iliac vessels at the pelvic brim. The right ureter is more frequently affected because it crosses the iliac vessels more distally than the left ureter. Additionally, the left ureter is relatively protected by the position of the uterus (dextro-rotation) and the presence of the sigmoid colon.^12^ Hormonal effects, particularly the smooth muscle relaxation induced by progesterone, also contribute to ureteral dilation during pregnancy.^11^

Underactive bladder (UAB) was identified as the fourth most common cause of hydronephrosis in the current study, accounting for 5.7% of cases. UAB remains a poorly understood condition characterized by impaired detrusor muscle contractility. Clinically, its present symptoms such as slow- or intermittent urinary flow, hesitancy, straining, or incomplete emptying. The etiology of UAB can be classified into several categories: neurogenic causes, such as cerebrovascular accidents; myogenic causes, such as bladder outlet obstruction; idiopathic cause, such as age-related degeneration; and iatrogenic causes, including the use of certain medications.^13^ In addition to UAB, other obstructive causes of hydronephrosis include congenital anomalies such as ureteropelvic junction obstruction^14^ and posterior urethral valves (PUV), which are commonly identified in antenatal hydronephrosis.^15^ Acquired obstructive causes of hydronephrosis include tumors,^16^ ureteral strictures,^17^ infections^18^ and ureterocele.^19^ Another important yet rare cause is idiopathic retroperitoneal fibrosis which can result in ureteral obstruction and, if not diagnosed and treated promptly, may lead to irreversible kidney damage.^20^

Anatomically, the ureter is divided into five segments: the pelviureteric junction (PUJ), the upper ureter (UU), located cranial to the sacroiliac joint (SIJ), the middle ureter (MU), overlying the SIJ, the distal ureter (DU), located distal to the SIJ and the ureterovesical junction (UVJ).^21^ In the current study, the most common site of the stones causing hydronephrosis was the DU, accounting for 43.7%. This finding is compatible with several previous studies. Bishnu et al. reported that the majority of ureteric stones were found in DU, followed by PUJ, with the fewest in the UVJ [7]. Similarly, Alshoabi et al. found that 28.8% of stones were in the DU.^3^ Nery et al. reported that 72.1% of the ureteric stones were in the distal third of the ureter.^22^

In contrast, a research study by Song et al. reported that 46.3% of ureteric stones were found in the UVJ, followed by 30.5% in the UU and 16.8% in the DU.^23^ This finding may be attributed to the presence of two physiological narrowing points of the ureter, in the UU and in the UVJ.^24^ Additionally, the three main anatomical narrowing points in the ureter have been described: the PUJ, the point where the ureter pass anterior to the iliac vessels and the UVJ.^25^ Our results also demonstrated a positive correlation between stone size and the grade of hydronephrosis, with the mean stone size increasing as the grade of hydronephrosis progressed. This finding is compatible with a previous study by Alshoabi et al., who reported that stone size increased from Grade-II to Grade-IV hydronephrosis.^3^

Similarly, Song et al. found that stone diameter related to hydronephrosis grade.^26^ This relationship can be explained by intuitive understanding that larger stones are more likely to obstruct urinary flow, thereby leading to more severe hydronephrosis.

### Limitation:

This study was limited by its single-center design with relatively small sample size which may not fully represent the complete spectrum of hydronephrosis causes. As a result, some rare causes of hydronephrosis were not reported. Future research involving multicenter studies with large sample size is recommended to provide a more comprehensive understanding of both common and uncommon causes of hydronephrosis.

## CONCLUSION

This study concluded that nephrolithiasis was the predominant cause of hydronephrosis in the western region of Yemen, followed by vesicoureteral reflux, pregnancy and underactive bladder. Less frequent causes included ureteric stricture and infection. Nephrolithiasis were most located in the DU, MU and UU. Grade-II hydronephrosis was the most frequently observed. A strong correlation was found between stone size and grade of hydronephrosis, with the mean size of the stone increasing progressively from Grade-I to Grade-IV.

### Authors’ contribution:

**SAA:** Conceptualization, data analysis, prepared the manuscript. **AM:** Conducted ultrasonography examination, data collection and interpretation. **HFM:** Revised the clinical information of the manuscript. **AFA:** Edited language and revised the manuscript. **FHA and AMH:** Revised the manuscript. All authors have read and agreed to the final version of the manuscript to be published.

### Abbreviations

**PUJ:** Pelviureteric junction.

**UU:** Upper ureter.

**MU:** Middle ureter.

**DU:** Distal ureter.

**UVJ:** Ureterovesical junction.

**ANOVA:** Analysis of variance.

**SPSS:** Statistical package for the social sciences.
